# Laser speckle imaging for visualization of hidden effects for early detection of antibacterial susceptibility in disc diffusion tests

**DOI:** 10.3389/fmicb.2023.1221134

**Published:** 2023-06-29

**Authors:** Ilya Balmages, Aigars Reinis, Svjatoslavs Kistkins, Dmitrijs Bliznuks, Emilija Vija Plorina, Alexey Lihachev, Ilze Lihacova

**Affiliations:** ^1^Biophotonics Laboratory, Institute of Atomic Physics and Spectroscopy, University of Latvia, Riga, Latvia; ^2^Institute of Computer Control, Automation and Computer Engineering, Riga Technical University, Riga, Latvia; ^3^Pauls Stradins Clinical University Hospital, Riga, Latvia; ^4^Department of Biology and Microbiology, Riga Stradins University, Riga, Latvia

**Keywords:** phenotypic antibacterial resistance, antibacterial resistance estimation, laser speckle imaging, sub-pixel correlation analysis, image processing, disc diffusion method

## Abstract

Rapid identification of effective antibiotic treatment is crucial for increasing patient survival and preventing the formation of new antibiotic-resistant bacteria due to preventative antibiotic use. Currently utilized “gold standard” methods require 16–24 h to determine the most appropriate antibiotic for the patient’s treatment. The proposed technique of laser speckle imaging with subpixel correlation analysis allows for identifying dynamics and changes in the zone of inhibition, which are impossible to observe with classical methods. Furthermore, it obtains the resulting zone of inhibition diameter earlier than the disk diffusion method which is recommended by the European Committee on Antimicrobial Susceptibility Testing (EUCAST). These results could improve mathematical models of changes in the diameter of the zone of inhibition around the disc containing the antimicrobial agent, thereby speeding up and facilitating epidemiological analysis.

## 1. Introduction

Rapid identification of antibacterial resistance plays a crucial role in treating acute infectious conditions such as sepsis and others. Empiric antimicrobial treatment must be replaced by pathogen-directed therapy as soon as possible to reduce patient mortality risk. Early identification of the pathogen and determination of its susceptibility to antibiotics can provide targeted pharmacological intervention at the early stages of the disease, increasing patient survival chances. This issue has become particularly relevant in treating elderly and immunocompromised patients. The EUCAST-standardized phenotypic resistance tests, such as disk diffusion and E-test, require 16–24 h to obtain results (Tenover, 2019). At the same time, despite the widespread use in detection of microorganisms by PCR method, the tests provides only genotypic information about antibacterial resistance, having limitations in interpretation of antibiotic susceptibility results in gram-negative pathogens (Bard and Lee, 2018). Therefore, new methods are needed that will allow for detecting changes in microorganisms faster than existing methods, significantly improving epidemiological work. Developing new, cost-effective methods for evaluating microbial activity to reduce detection time is in full compliance with the World Health Organization’s (WHO) published Global Action Plan (World Health Organization, 2020). This is of great practical interest to technology developers and scientists.

In previous studies using a non-contact optical technique called laser speckle contrast imaging, we determined bacterial growth after 3 h from the beginning of activity (Balmages et al., 2021b). In our microbiology experiments performed on solid media the term “activity” refers to a wide range of environmental changes including bacteria doubling, surface roughness changes caused by colony growth, as well as fluctuations of optical properties of the media caused by bacteria growing process (Balmages et al., 2023). A laser speckle is an interference pattern produced by coherent light reflected from an illuminated rough surface. If the reflected surface is stationary, the scattered light creates constant patterns of laser speckles. However, if there are changes on the surface the individual speckle appears to “twinkle.” This is called a “time-varying speckle.” This method has been proven to track moving particles in optically inhomogeneous media by analyzing time-varying laser speckle patterns. In the laser speckle imaging method, a laser beam is scattered on a Petri dish where the test bacteria and antibiotic discs are located. The laser speckles reflected from the surface are recorded sequentially in time. Subpixel correlation analysis was proposed to detect small changes in the sequence of laser speckle images, and the effects associated with changes in bacterial activity can be observed.

We suppose that the laser speckle technique, combined with subpixel correlation analysis, can provide a sensitive and early detection method for assessing bacterial growth inhibition and the formation of a sterile zone in the presence of antibiotics. For this reason, the aim of the study is to assess the feasibility and effectiveness of the laser speckle technique combined with subpixel correlation analysis for detecting bacterial growth inhibition and the formation of a zone of inhibition in the presence of antibiotics. Additionally, the study aims to evaluate the spatiotemporal characteristics of the formation of zone of inhibition and compare the performance of the laser speckle technique with traditional methods for assessing bacterial growth inhibition.

This study demonstrates the advantages of the proposed method - faster antimicrobial susceptibility results and, therefore, faster replacement of empiric antimicrobial therapy with pathogen-targeted therapy, which has greater potential to reduce treatment duration and costs, as well as to reduce mortality.

## 2. Materials and methods

Experiments were performed at the Pauls Stradins Clinical University Hospital Joint Laboratory on different bacteria and their corresponding antibiotics (Karlowsky et al., 2003).

### 2.1. The experimental setup

**Figure 1:**
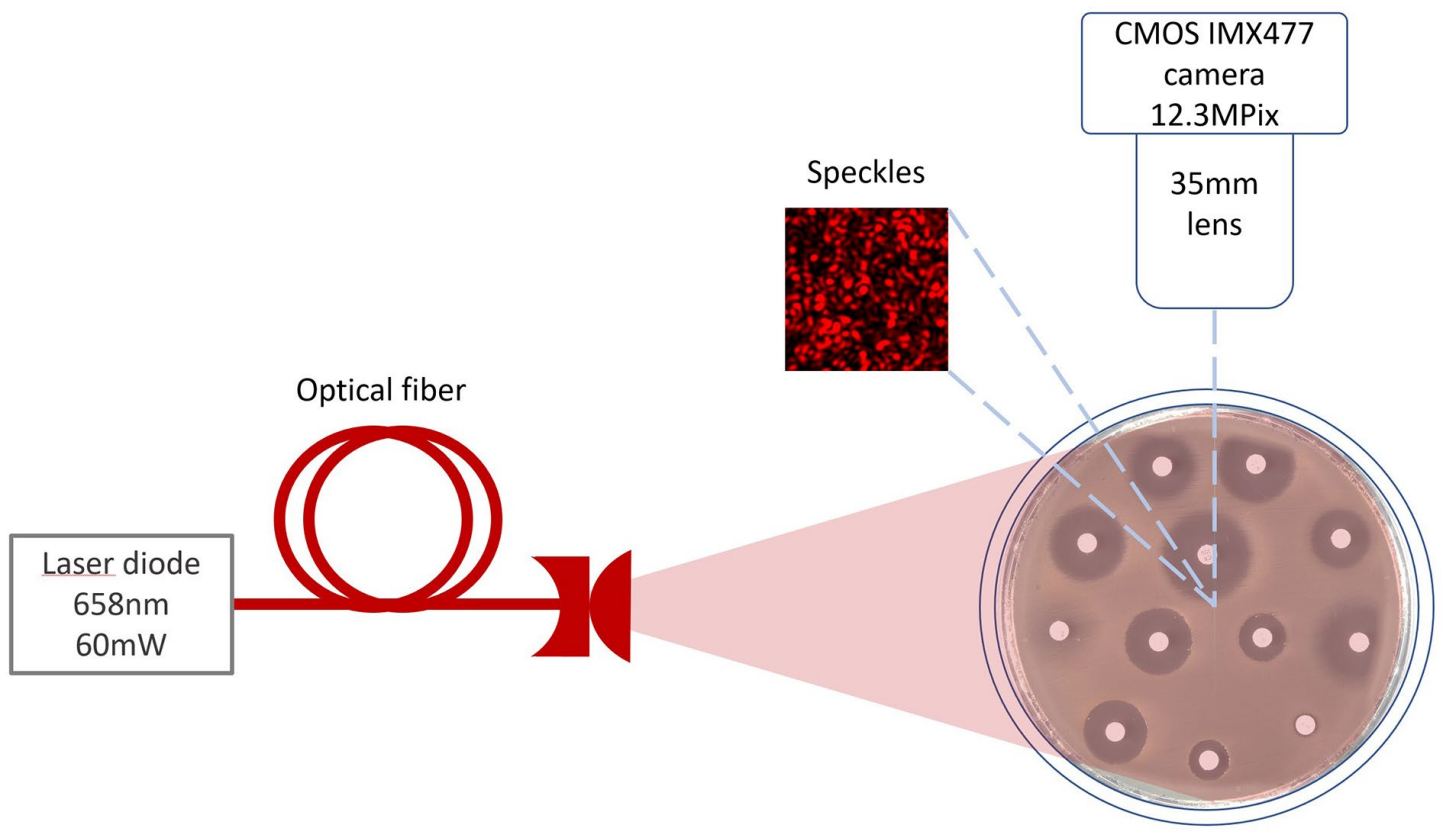
Setup scheme for burst image capturing of bacteria growing process under 658 nm laser illumination.

The laser speckles were generated by a single mode 658 nm diode-pumped solid-state laser (output power 60 mW). Images were captured at 20-s intervals by a CMOS camera (Figure 1). To read the inscriptions on the antibiotic discs, an RGB image was captured under white LED illumination.

### 2.2. Microbial strains and cultivation conditions

Clinical isolates of *Escherichia coli* (*E. coli*) and *Klebsiella aerogenes* (*K. aerogenes*) bacteria were selected for the experiment. We chose cefotaxime disk (CTX, 5 μg) and ampicillin disk (AM, 10 μg) for *K. aerogenes* and ciprofloxacin disk (CIP, 5 μg) for *E. coli*, and amikacin disk (AK, 30 μg), for an experiment without bacteria. To avoid artifacts, the experiments were performed in a separate room, in an incubator at 37°C. The bacterial suspensions were made in saline to the density of a 0.5 McFarland turbidity standard. Culturing on the Petri dish was prepared according to a EUCAST standard procedure - bacteria were inoculated on Mueller-Hinton agar and then antibiotic discs were placed on the surface (The European Committee on Antimicrobial Susceptibility Testing, 2023). To estimate how the diffusion of antibiotics, drying of the agar surface and other artifacts influence the observed signal, Petri dishes with agar and antibiotics (without bacteria) were prepared for reference measurements.

### 2.3. Laser speckle image conversion to time signals

The images captured at 20 s intervals were processed by dividing the experimental field into small sections of NxN pixels. A two-dimensional normalized cross-correlation was performed between consecutive NxN image fragments throughout the experiment (Gåsvik, 2002).


(1) $$\begin{array}{l} NCC\left(u,v\right)=\\ \frac{\sum_{x}\sum_{y}\left(\left(a\left(x,y\right)-\bar{a}\right)\cdot \left(b\left(x-u,y-v\right)-\bar{b}\right)\right)}{\sqrt{\sum_{x}\sum_{y}{\left(a\left(x,y\right)-\bar{a}\right)}^{2}\cdot \sum_{x}\sum_{y}{\left(b\left(x-u,y-v\right)-\bar{b}\right)}^{2}}} \end{array}$$

where a(x, y) and b(x, y) are two adjacent frames in the sequence, $\bar{a}$ and $\bar{b}$ are the average values of these two frames, *u* and *v* are spatial displacements between frames a(x, y) and b(x, y) toward x and y, respectively.

The correlation peak shift in space characterizes the changes that occur between consecutive images.


(2) $$\left(\hat{u},\hat{v}\right)=\underset{u,v}{\arg\max}\left[NCC\left(u,v\right)\right]$$

Interpolation around the correlation peak is performed to find a more accurate peak position (Lai and Torp, 1999).


(3) $${\hat{\delta }}_{x}=-\frac{b_{u}}{2a_{u}}=\frac{NCC\left(\hat{u}-1,\hat{v}\right)-NCC\left(\hat{u}+1,\hat{v}\right)}{2\left(NCC\left(\hat{u}-1,\hat{v}\right)-2NCC\left(\hat{u},\hat{v}\right)+NCC\left(\hat{u}+1,\hat{v}\right)\right)}$$

where $a_{u}$ and $b_{u}$ are the parabolic coefficients.

The offsets obtained between each pair of adjacent images were accumulated and converted into a “time signal.”


(4) $$sig\;\left[n\right]=\overset{n}{\underset{i=1}{\sum }}\hat{\delta }\left[i\right]$$

To avoid the influence of local transient spikes, a signal envelope within a certain window was used (Tukey, 1974).


(5) $$Env\left[n\right]=\sqrt{\frac{1}{N}\sum_{k=n-N+1}^{n}sig{\left[k\right]}^{2}}$$

where N is the length of the window, n is the current sample, k is the index running inside the window. Accordingly, N – the length of the window – is responsible for the degree of signal smoothing.

An increase of signal values represents spatiotemporal activity patterns during colony growth (Balmages et al., 2023).

## 3. Results

### 3.1. Comparison of the results obtained with the laser speckle technique without and with the algorithm

**Figure 2:**
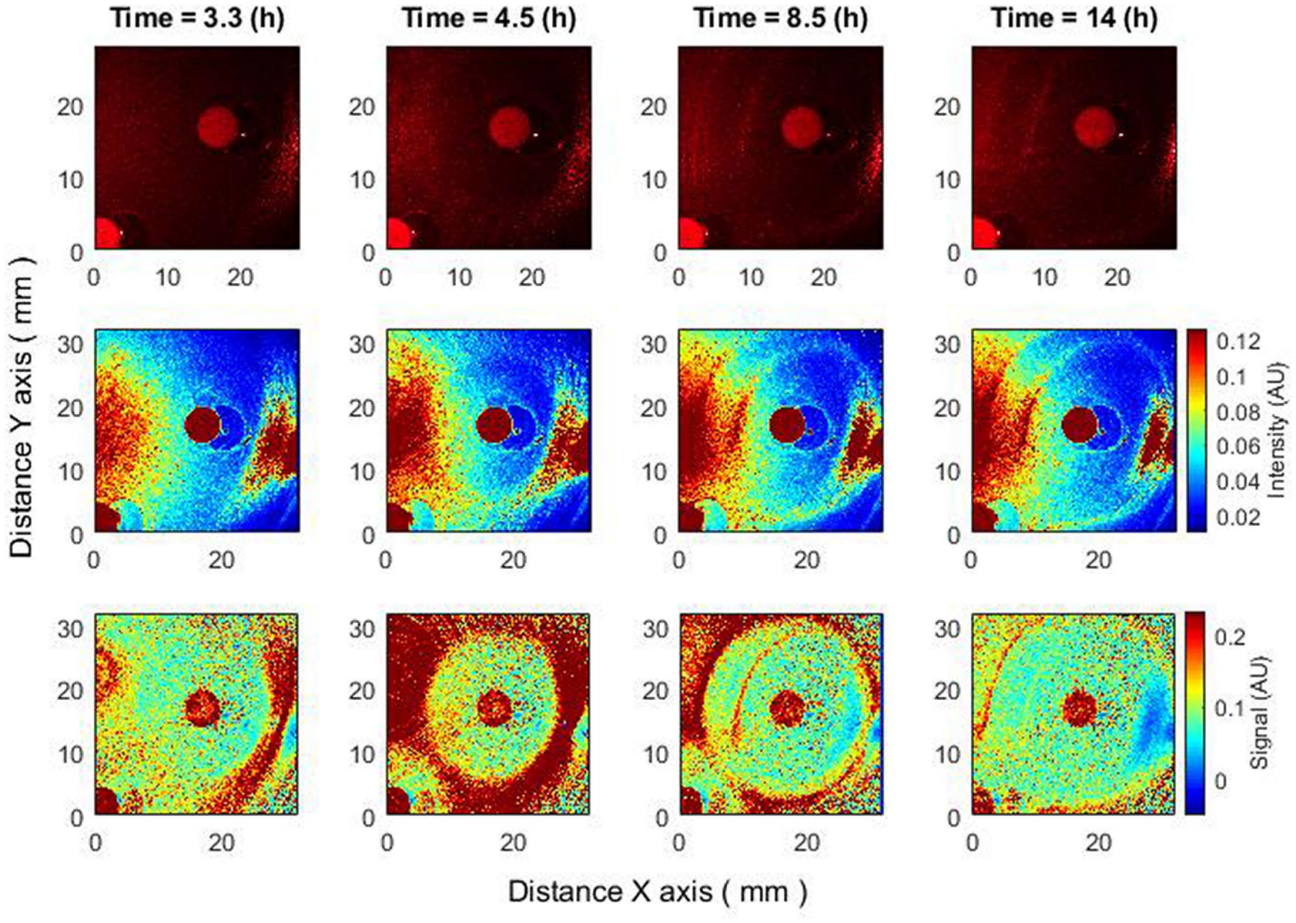
Changes of zone of inhibition as a function of time. Bacteria: *E. coli*, antibiotic CIP 5 µg. Recorded raw laser speckle images in time **(top row)**, records processed by smoothing filter, and contrast enhancements **(middle row)** and resulting images after the subpixel correlation analysis **(bottom row)**.

**Figure 3:**
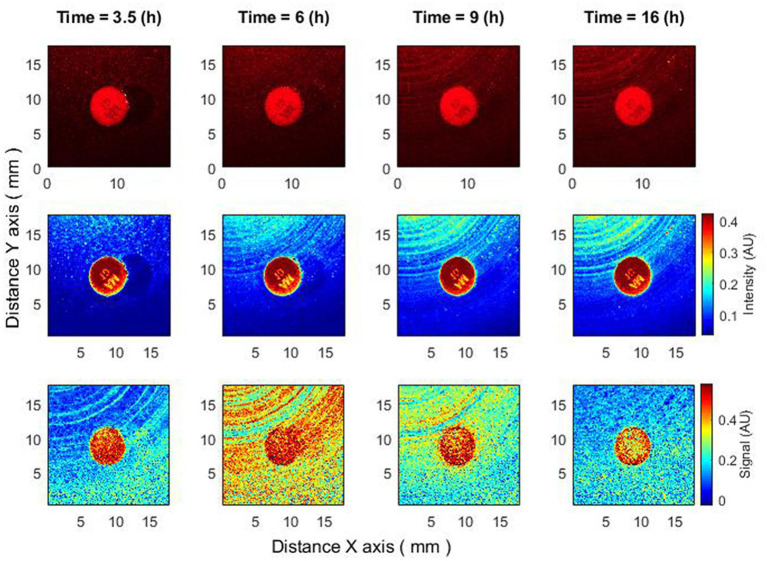
The behavior of bacteria that are resistant to antibiotics. Bacteria: *K. aerogenes*, antibiotic AM 10 µg. Recorded raw laser speckle images in time **(top row)**, records processed by smoothing filter, and contrast enhancements **(middle row)** and resulting images after the subpixel correlation analysis **(bottom row)**.

In this section, we compare the behavior of the zone of inhibition for raw speckle images and for speckle images processed using subpixel correlation analysis. Two cases of bacterial susceptibility were considered: (1) bacteria that are susceptible to the antibiotic (Figure 2) and (2) bacteria that are resistant to the antibiotic (Figure 3).

The subpixel correlation technique allows for the detection of submicron bacterial events (e.g., the formation of a zone of inhibition) earlier than without the algorithm (Figure 2). It can be observed that by performing the subpixel correlation algorithm in the given experiment, the zone of inhibition can be detected after 3.3 h, but without executing the algorithm, the zone of inhibition can be detected no earlier than after 4.5 or more hours.

The proposed subpixel correlation algorithm allows it to detect effects that are not observed without it (Balmages et al., 2021a, 2023).

**Figure 4:**
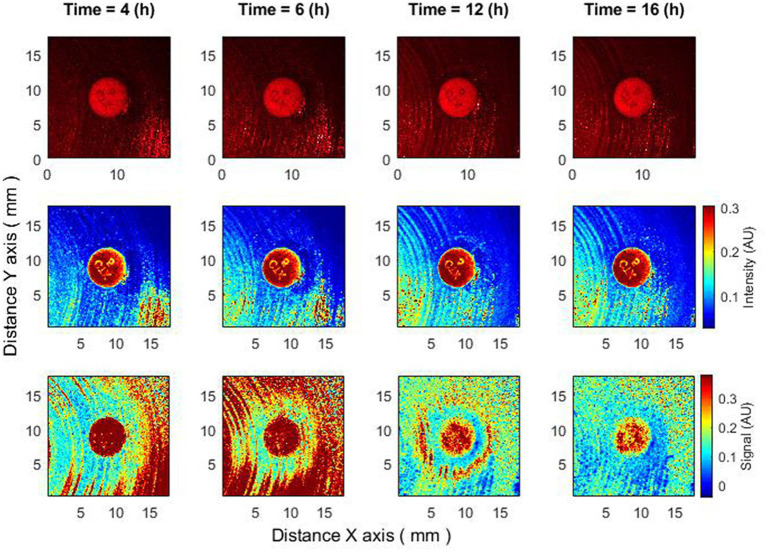
The behavior of bacteria on raw speckle images does not demonstrate the zone of inhibition, while the subpixel correlation analysis demonstrates the formation of a zone of inhibition. Bacteria: *K. aerogenes*, antibiotic CTX 5 µg. Recorded raw laser speckle images in time **(top row)**, records processed by smoothing filter, and contrast enhancements **(middle row)** and resulting images after the subpixel correlation analysis **(bottom row)**.

**Figure 5:**
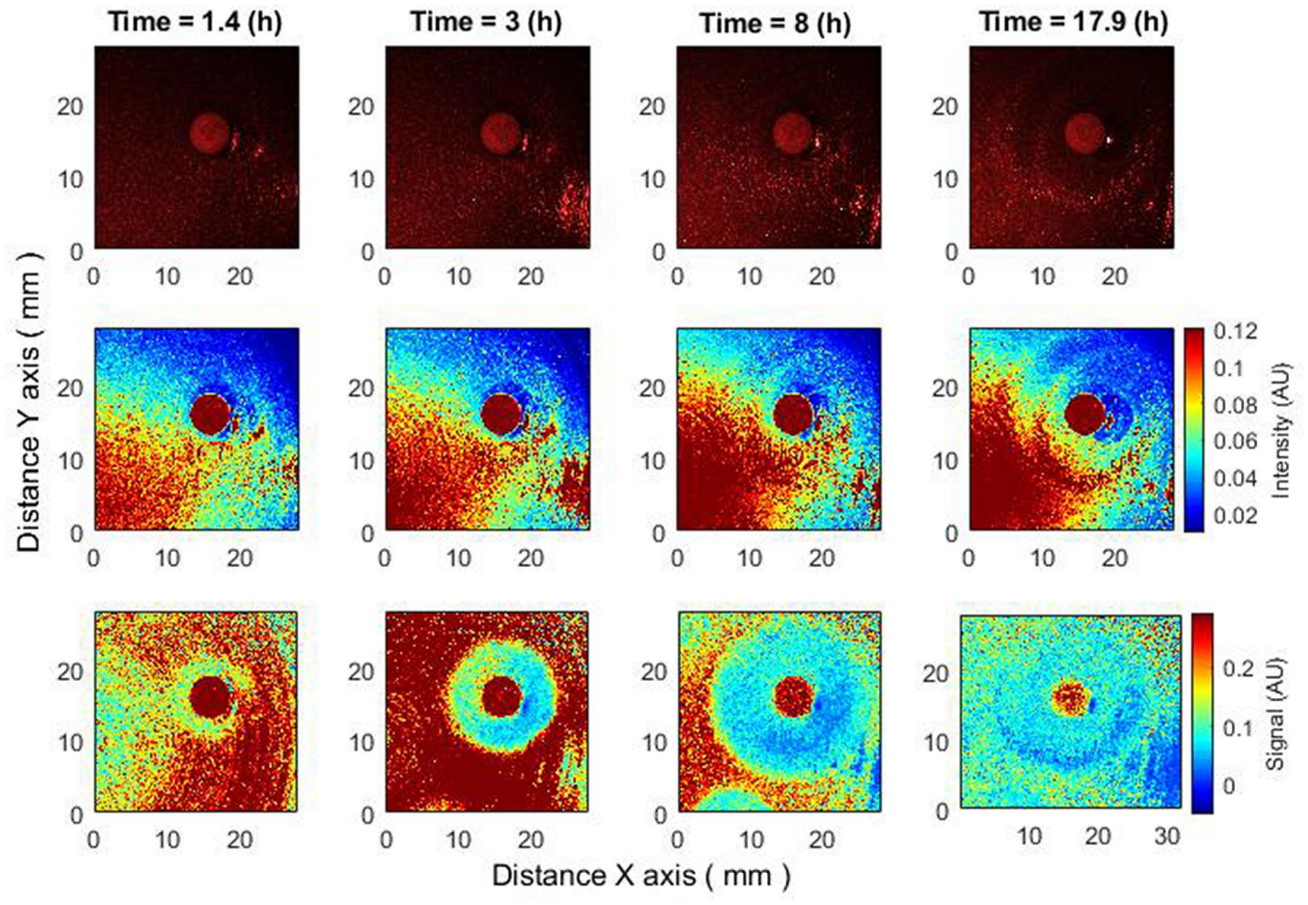
Antibiotics are placed 4–4.5 h after bacteria inoculation. Zone of inhibition changes as a function of time. Bacteria: *E. coli*, antibiotic CIP 5 µg. Recorded raw laser speckle images in time **(top row)**, records processed by smoothing filter, and contrast enhancements **(middle row)** and resulting images after the subpixel correlation analysis **(bottom row)**.

**Figure 6:**
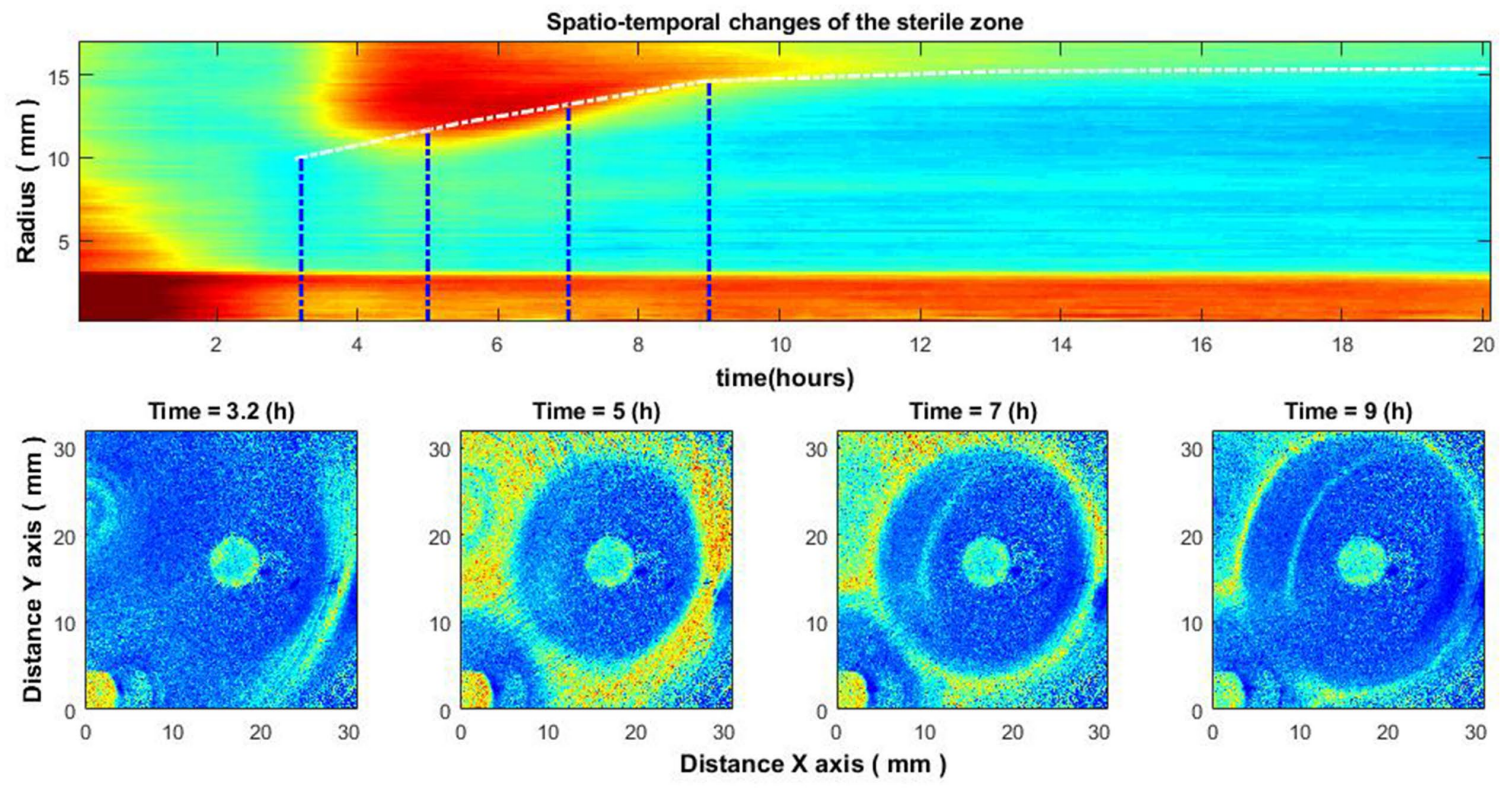
Spatio-temporal changes of the zone of inhibition **(top)**, and zone of inhibition formation in the growth of bacteria around the antibiotic disc **(bottom)**. Bacteria: *E. coli*, antibiotic CIP 5 µg. Antibiotics were placed on the Petri dish immediately after bacteria inoculation.

**Figure 7:**
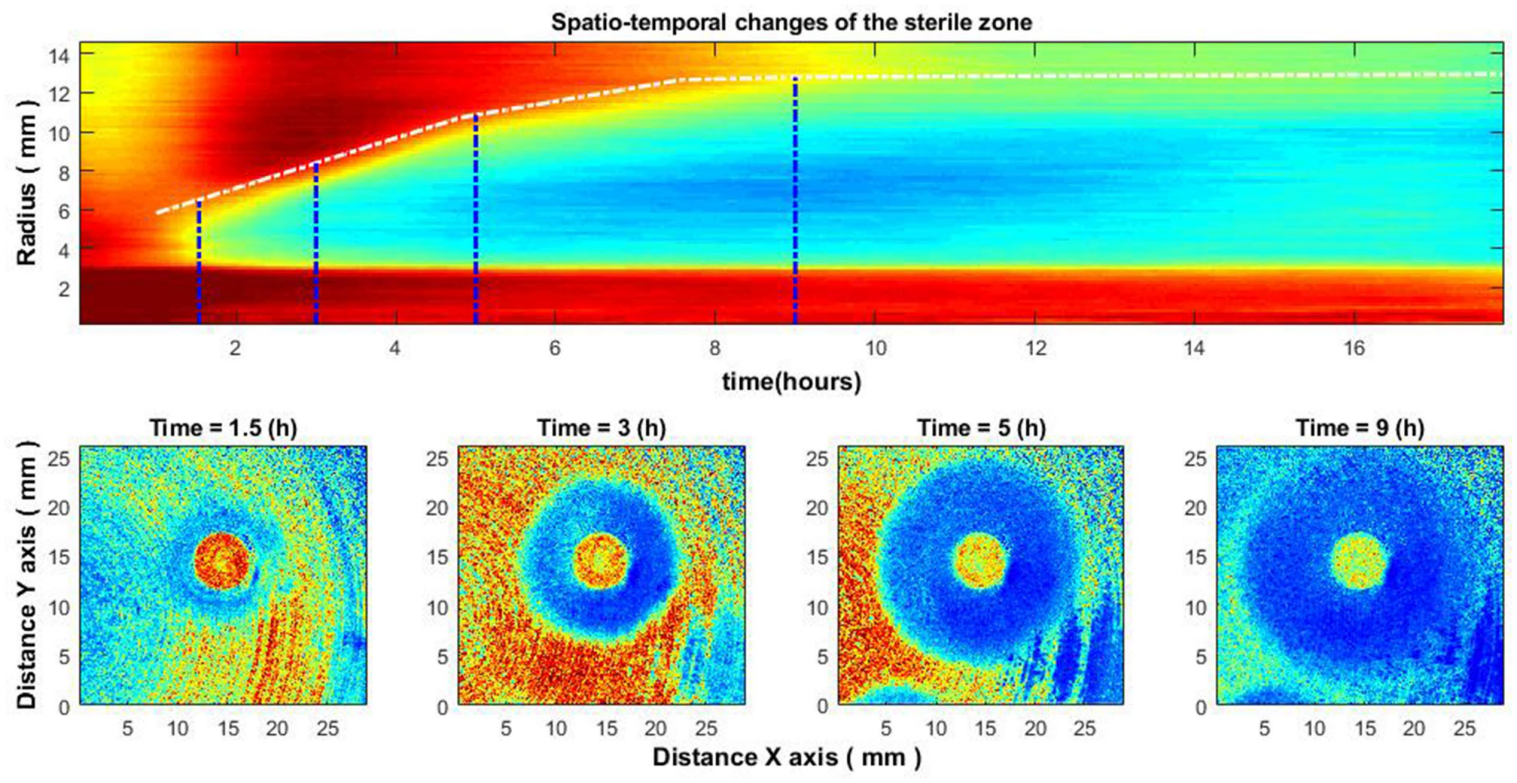
Spatio-temporal changes of the zone of inhibition **(top)**, and zone of inhibition formation in the growth of bacteria around the antibiotic disc **(bottom)**. Bacteria: *E. coli*, antibiotic CIP 5 µg. Antibiotics were placed on the Petri dish 4–4.5 h after the bacteria inoculation.

**Figure 8:**
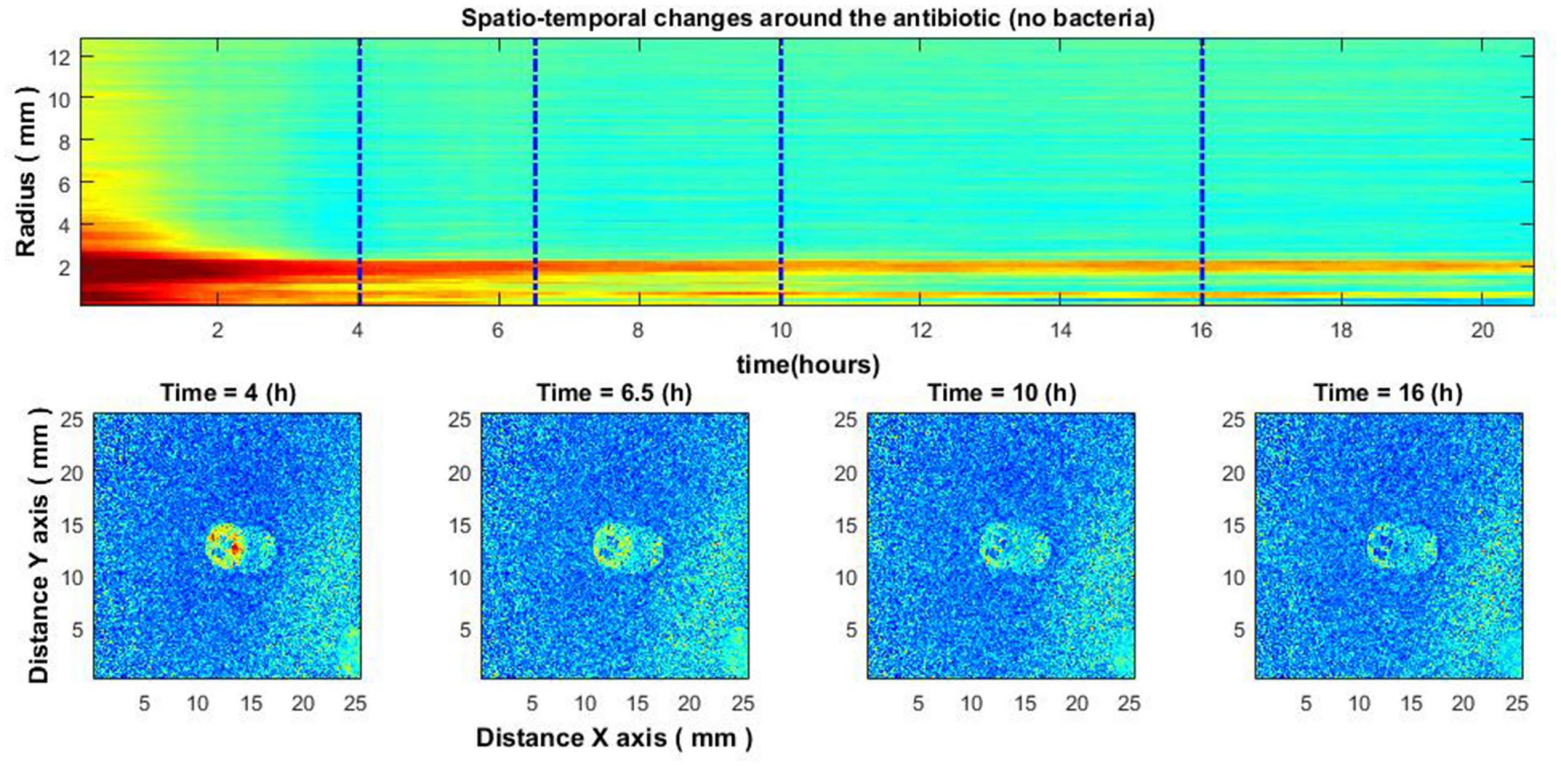
Experiment without bacteria. Spatio-temporal image of the area around the antibiotic AK 30 µg **(top)** and the spatial zone around the antibiotic at different times **(bottom)**.

When the bacteria are completely resistant to the antibiotic, raw speckle images and speckle images after subpixel correlation analysis will show that the zone of inhibition is not observed. However, since subpixel correlation analysis is designed to detect activity, there will be noticeable differences in bacterial behavior. Raw speckle images do not yet demonstrate the presence of bacteria (3.5 h). Speckle images after subpixel correlation already detect it. That is, the activity of bacteria already exists. In the images showing data analysis using subpixel correlation analysis (i.e., Figures 2–5, bottom row and Figures 6–8 both top and bottom row), blue color indicates low spatiotemporal activity, while red color indicates high bacterial activity. It is also worth noting that the antibiotic is in red color, that is, some processes take place on it, but these are chemical processes not related to the behavior of bacteria. The second interesting result is that raw speckle images still show the presence of spatiotemporal activity at late times (16 h), while speckle images after subpixel correlation do not detect them. This suggests that the bacteria may be in the plateau phase, where the equilibrium of duplication and cell death persists (Figure 3).

Another intriguing outcome can be observed when the bacteria are completely resistant to the antibiotic based on raw speckle images and the zone of inhibition is not observed at all, while the subpixel correlation analysis demonstrates the formation of a zone of inhibition (Figure 4). This indicates that in some situations the zone of inhibition may form even if it is not detected by the “naked eye” at all and the described method is able to detect such situations.

### 3.2. Early detection of the formation of zone of inhibition

In cases where a zone of inhibition is formed, it becomes noticeable after 3–4 h from the beginning of the experiment. It appears “suddenly,” immediately possessing a certain radius. The question arises: what processes cause this sudden zone to appear? New experiments were conducted to better understand these processes. In previous experiments, bacteria were inoculated on agar medium in a Petri dish, and antibiotic discs were immediately placed on the prepared surface. In the new experiments, the antibiotic discs were placed on the surface 4–4.5 h after the bacteria inoculation.

We assume that there is no more bacterial activity in the zone of inhibition when the signal level acquired from the subpixel correlation method processing is significantly (several times) lower than the signal from the zone where bacterial activity occurs. In the zone where bacterial activity occurs, we get increased signal values. Bacterial activity using the subpixel correlation method can be detected 3–4 h after inoculation (Balmages et al., 2020, 2021a,b). Until then, the signal level is very low. The zone of inhibition is noticeable only in contrast to the activity of bacteria; accordingly, it also cannot be visible until this moment. Since after 3.5–4 h, the bacteria have grown to a sufficient concentration to obtain a signal from bacterial activity with the established method, we expected that application of antibiotic discs 4–4.5 h after bacterial inoculation would immediately allow us to obtain the formation of the zone of inhibition.

This effect can be observed in a signal proportional to activity, i.e., after subpixel correlation analysis. If we observe raw speckle images, then this effect may not be noticeable since it is not the activity of bacteria that is observed there, but their presence. Bacteria are already present for several hours, and when the antibiotic disk is placed, they begin to “lose” activity. Hence, raw speckle images still do not demonstrate the formation of a zone of inhibition, while it is observed using subpixel correlation analysis of laser speckle images (Figure 5).

Thus, using subpixel correlation analysis of laser speckle images, it is possible to measure the formation of a zone of inhibition already 1–1.5 h after the time when the antibiotic was placed on already grown bacterial cultures. And what is important – the zone of inhibition did not appear “suddenly,” but was formed with a small radius, continuously increasing, which can be explained by the spread of the antibiotic in the agar.

### 3.3. Spatiotemporal analysis of the changes of zone of inhibition

It was obtained that after a few hours from the start of the experiment, a disc of the zone of inhibition begins to form around the antibiotic. The zone of inhibition becomes clearly visible within a couple of hours after the beginning of formation. The result was obtained significantly earlier than by the disk diffusion method for the same bacterial species (The European Committee on Antimicrobial Susceptibility Testing, 2023). The formation of a zone of inhibition is characterized by a decrease in activity. That is, we are looking for a drop (occurrences of low values after peaks of signal envelope). It should be considered that detecting low values of signal in noisy environments is more difficult than detecting high values. To avoid the influence of noise, it is worth averaging the signal envelopes over each radius as it moves away from the center (from the antibiotic). Consider one averaged signal envelope for each radius.


(6) $$\bar{Env\left[r,n\right]}=\left[\frac{1}{M_{R1}}\overset{M_{R1}}{\underset{m_{R1=1}}{\sum }}Env_{m_{R1}}\left[n\right],\ldots ,\frac{1}{M_{Rk}}\overset{M_{Rk}}{\underset{m_{Rk=1}}{\sum }}Env_{m_{Rk}}\left[n\right]\right]$$

Where $M_{Rk}$ is the number of envelope signals at a given distance/radius from the center.

**Figure 9:**
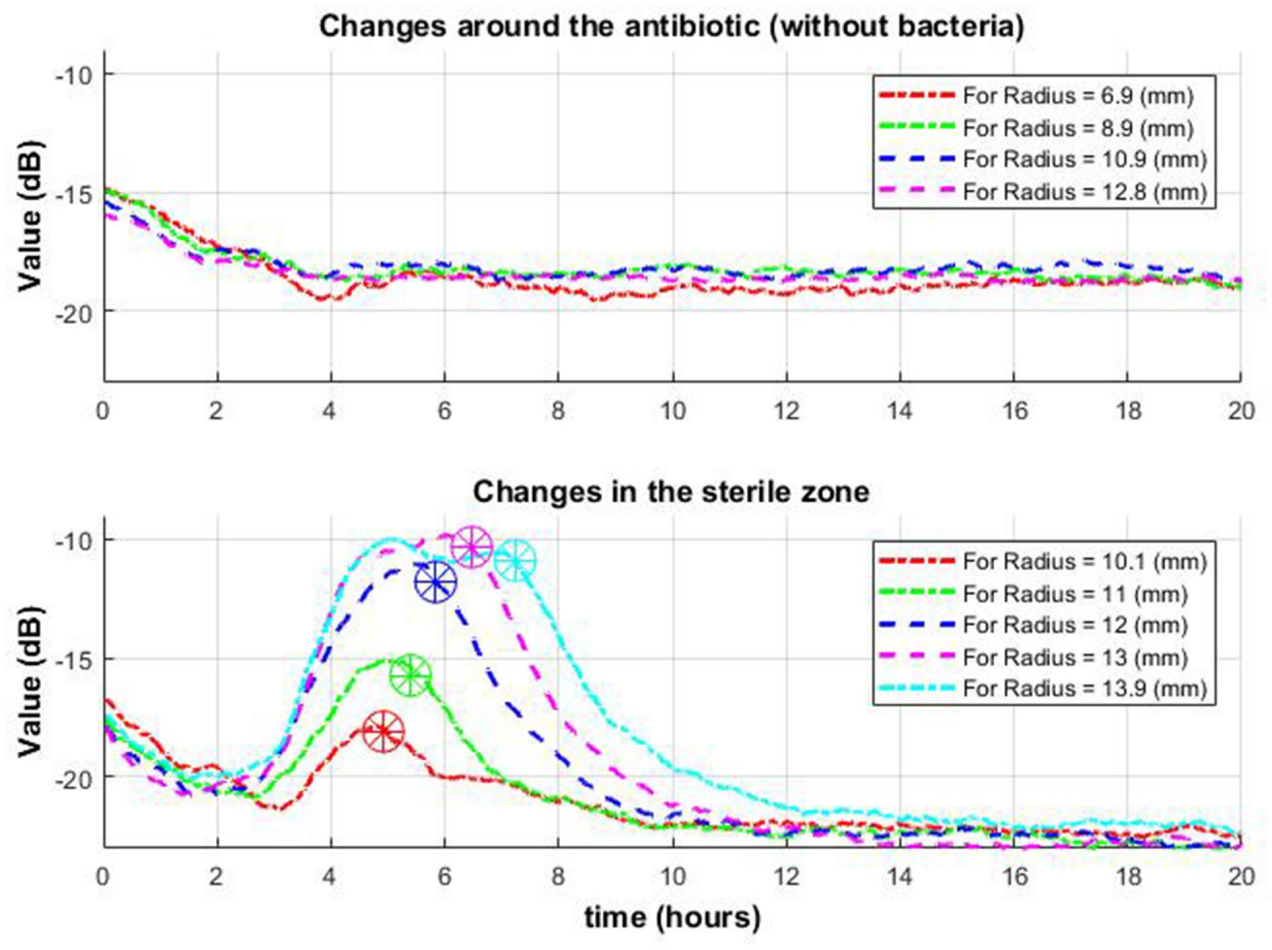
Change of signal envelope over time for several different radii around the antibiotic: without bacteria **(top row)** and with bacteria **(bottom row)**. On the bottom graph, it is observed that as it moves away from the center, the drop (which means the appearance of a zone of inhibition in this place) occurs later (stars in a circle).

Consider the time when the signal envelope starts to drop as a function of time for several received radii. Note that the beginning of signal envelope drop might indicate the appearance of a zone of inhibition in this place. Whereas in the situation where antibiotics are placed without bacteria such behavior was not found (Figure 9).

Putting the averaged signal envelopes for all radii together, we get a spatio-temporal image. By creating this image, we can study the behavior of the zone of inhibition. By emphasizing the drop for each radius, we can obtain a certain curve, according to which the size of the zone of inhibition changes over time (Figure 6). This behavior has been observed in several different experiments for different species of bacteria using different antibiotics.

Figure 6 illustrates the case of bacteria susceptible to antibiotics. Antibiotics were placed on the Petri dish immediately after the bacteria inoculation. It can be observed that there is no space–time reaction before 3.5–4 h. This is due to the reason described above – the activity of bacteria is not noticeable; accordingly, the zone of inhibition is not visible. Consider similar images when the antibiotic was placed on the Petri dish 4–4.5 h after the bacteria inoculation (Figure 7). Then there is no such “gap,” and the spatio-temporal image is continuous.

For reference, experiments were performed with antibiotic diffusion discs placed on agar without inoculated bacteria. In such cases, the zone of inhibition was not observed (Figure 8). This indicates that diffusion of the antibiotic without the presence of bacteria cannot be observed with our established method.

## 4. Summary and discussion

The potential of laser speckle imaging techniques for antimicrobial susceptibility testing have been earlier demonstrated by many authors (Grassi et al., 2016, 2019; Zhou et al., 2020). Authors described promising results obtained from variable laser speckle imaging analysis of bacteria growth kinetics in solid and liquid media under influence of different antibiotics. Results showed quite good sensitivity of the speckle imaging method for visualization of antibacterial effect on bacterial growth. However, literature data lacks exact procedures for practical clinical use and does not provide correlation with classical antimicrobial susceptibility testing (AST). Bacteria colony growth kinetics acquired by processing of speckle images under influence of antibiotics itself is not a clinically significant parameter and cannot be used for robust evaluation of susceptibility for a wide range of all tested drugs.

Similarly, as other rapid antimicrobial susceptibility tests (RAST) the technology has several limitations. Firstly, the slow growth rate of certain bacterial strains can prolong the time required for conducting AST, which may limit the feasibility of obtaining results within a desired timeframe. Secondly, the presence of heterogeneous antibiotic resistance within bacterial populations can affect the accuracy of AST results, as different subpopulations may exhibit varying levels of susceptibility or resistance. However, in the context of presented study and the technology developed, it is important to consider the clinical timepoints for administering antibiotics. Many broad-spectrum antibiotics are given at intervals of 6 to 8 h, allowing clinicians to reassess the choice of antibiotics based on the patient’s condition. Future advancements in identification and antibiotic susceptibility testing (ID/AST) methods should focus on providing results within this time frame of less than 6 h, enabling timely adjustments in antibiotic therapy to optimize patient care (Tjandra et al., 2022).

In this paper the clinically significant dynamics and changes of the zone of inhibition were evaluated by the processing of laser speckle images with the subpixel correlation analysis method. The zone of inhibition is the “absence of signal;” accordingly, it can be detected on the “signal presence” background, that is, only when the bacteria are already visible (after 3–4 h). The lag phase continues for up to 3–4 h, until the surface density of bacteria increases to the level necessary for observation. However, if the antibiotic is placed on the Petri dish 4–4.5 h after the bacteria inoculation, the bacteria are in sufficient concentration to be visible with the subpixel correlation method. In this case, we observe that the formation of the zone of inhibition is clearly visible almost immediately. While observing unprocessed (raw) speckle images in this case, the zone of inhibition is not immediately visible, but after a delay of several hours (approximately 5 h delay). A case was observed where bacteria determined to be fully resistant to antibiotics using the conventional method could show a formation of zone of inhibition when analyzed using subpixel correlation analysis on laser speckle images (Figure 4). This shows that the established method allows us to observe small changes in activity that cannot be observed in the raw data. Hence, when developing a novel assay that deviates significantly from the current reference standard, it is crucial to consider various factors that can influence antibiotic responses. Factors such as the potential inoculum effect, variations in bacterial growth phases, and delayed expression of induced resistance can significantly impact the assay’s performance. By incorporating an understanding of these factors into the design of the new assay, we can enhance its accuracy and reliability compared to the existing standard (Idelevich and Becker, 2019).

Our proposed technology on one hand would fit well into the clinical microbiology automation offered by any company, where the determination of antimicrobial susceptibility is based on the disk diffusion test, and on the other hand, it would help to save time and obtain antimicrobial susceptibility results faster.

## 5. Conclusion

In the present study, we have demonstrated the ability of subpixel analysis of laser speckle time series images for dynamic visualization of zones of inhibition produced by antimicrobials. The obtained results demonstrate the ability to follow changes in the radius of the zone of inhibition from early times when it cannot yet be detected by classical methods. Thus, this allows for a detailed analysis of the dynamics of the zone of inhibition, which in the future will make it possible to predict the diameter of the zone of inhibition around the antibiotic disk faster than using the disk diffusion method without laser speckle technology. The practical implementation of the obtained technology will allow the laboratory to send the results and their interpretation to the clinic much faster. This study will serve as a foundation for a deeper understanding of the zone of inhibition formation processes and the possibility of describing this zone using mathematical models, which will become the basis for further prediction of the zone of inhibition in our future studies.

## Data availability statement

The raw data supporting the conclusions of this article will be made available by the authors, without undue reservation.

## Author contributions

IB, AR, SK, DB, EP, AL, and IL: conceptualization, formal analysis, investigation and methodology, writing—review, and editing. AR, SK, DB, and EP: data curation. AR, SK, DB, AL, and IL: funding acquisition. AR, DB, AL, and IL: project administration. IB, AR, DB, and IL: resources. IB: software. IB, IL: supervision. IB and DB: visualization. IB, AR, and SK: writing—original draft. All authors contributed to the article and approved the submitted version.

## Funding

This work has been supported by the European Regional Development Fund project “Rapid assessment system of antibacterial resistance for patients with secondary bacterial infections” (No. 1.1.1.1/21/A/034).

## Conflict of interest

The authors declare that the research was conducted in the absence of any commercial or financial relationships that could be construed as a potential conflict of interest.

## Publisher’s note

All claims expressed in this article are solely those of the authors and do not necessarily represent those of their affiliated organizations, or those of the publisher, the editors and the reviewers. Any product that may be evaluated in this article, or claim that may be made by its manufacturer, is not guaranteed or endorsed by the publisher.
