# Corrigendum: Laser speckle imaging for visualization of hidden effects for early detection of antibacterial susceptibility in disc diffusion tests

**DOI:** 10.3389/fmicb.2023.1266723

**Published:** 2023-08-11

**Authors:** Ilya Balmages, Aigars Reinis, Svjatoslavs Kistkins, Dmitrijs Bliznuks, Emilija Vija Plorina, Alexey Lihachev, Ilze Lihacova

**Affiliations:** ^1^Biophotonics Laboratory, Institute of Atomic Physics and Spectroscopy, University of Latvia, Riga, Latvia; ^2^Institute of Computer Control, Automation and Computer Engineering, Riga Technical University, Riga, Latvia; ^3^Pauls Stradins Clinical University Hospital, Riga, Latvia; ^4^Department of Biology and Microbiology, Riga Stradins University, Riga, Latvia

**Keywords:** phenotypic antibacterial resistance, antibacterial resistance estimation, laser speckle imaging, sub-pixel correlation analysis, image processing, disc diffusion method

In the published article, the order of the image files for Figures 6, 7, 8, and 9 was incorrect. The corrected Figures 6, 7, 8, and 9, and their captions appear below.

**Figure 6:**
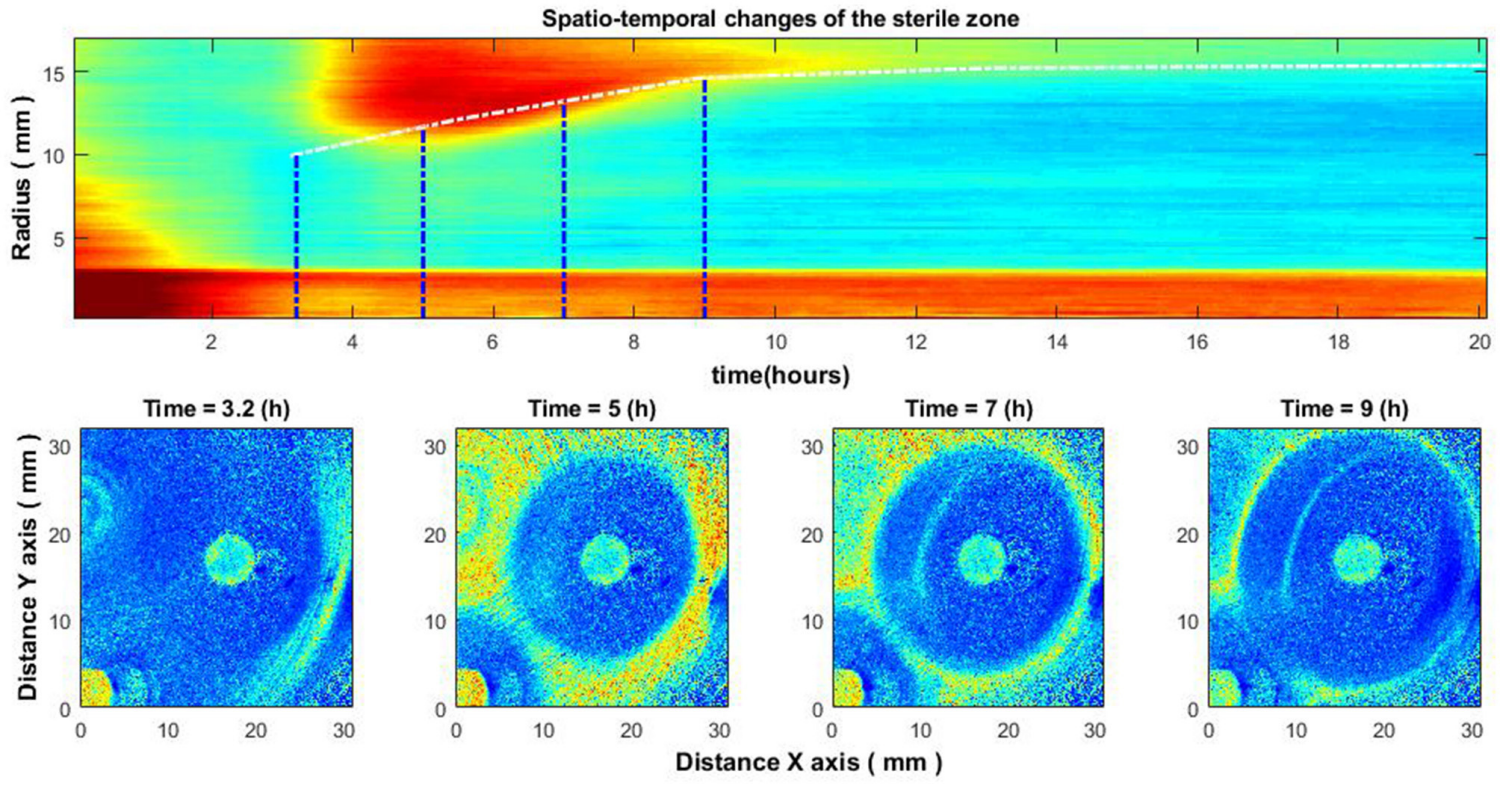
Spatio-temporal changes of the zone of inhibition **(top)**, and zone of inhibition formation in the growth of bacteria around the antibiotic disc **(bottom)**. Bacteria: *E. coli*, antibiotic CIP 5 μg. Antibiotics were placed on the Petri dish immediately after bacteria inoculation.

**Figure 7:**
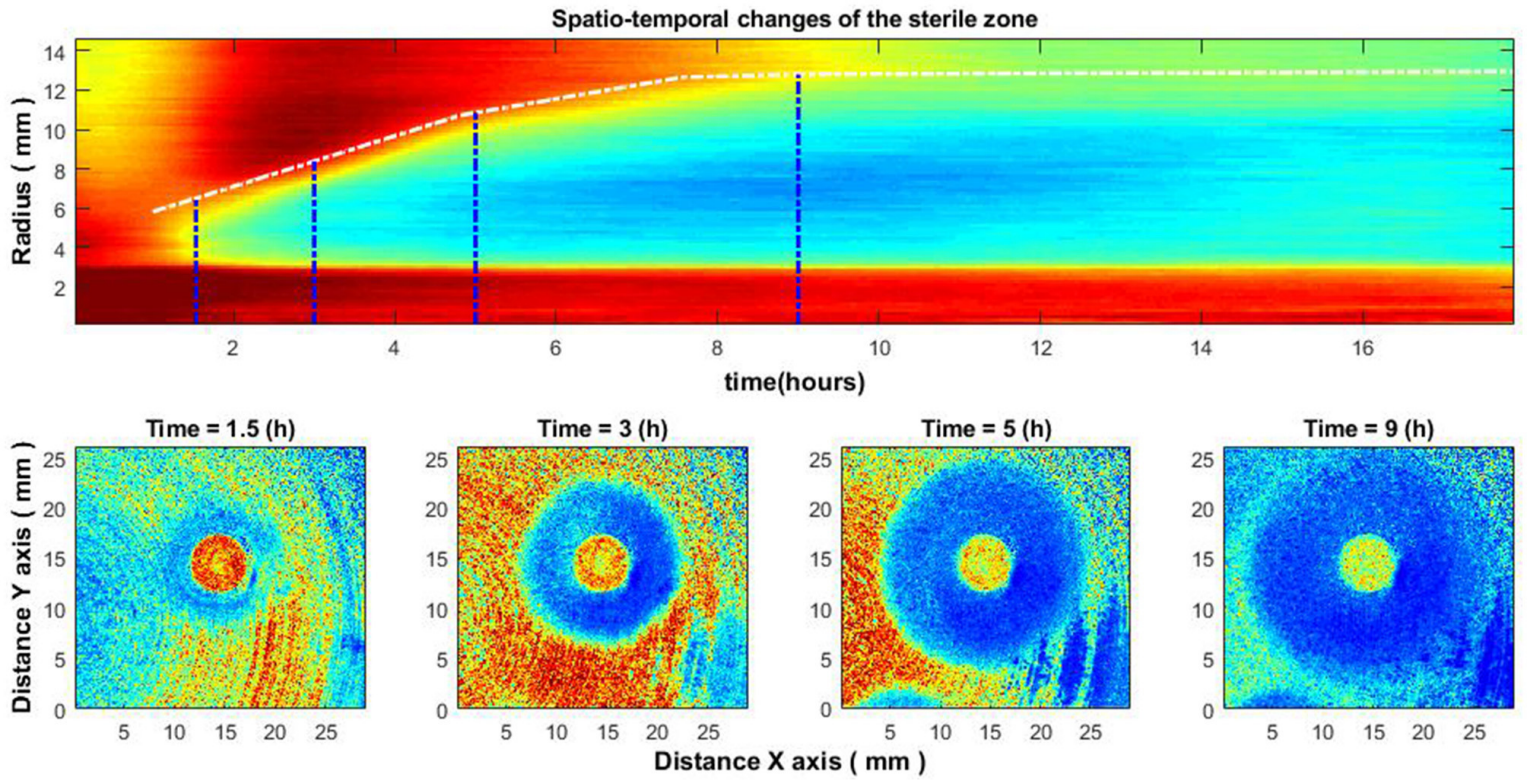
Spatio-temporal changes of the zone of inhibition **(top)**, and zone of inhibition formation in the growth of bacteria around the antibiotic disc **(bottom)**. Bacteria: *E. coli*, antibiotic CIP 5 μg. Antibiotics were placed on the Petri dish 4–4.5 h after the bacteria inoculation.

**Figure 8:**
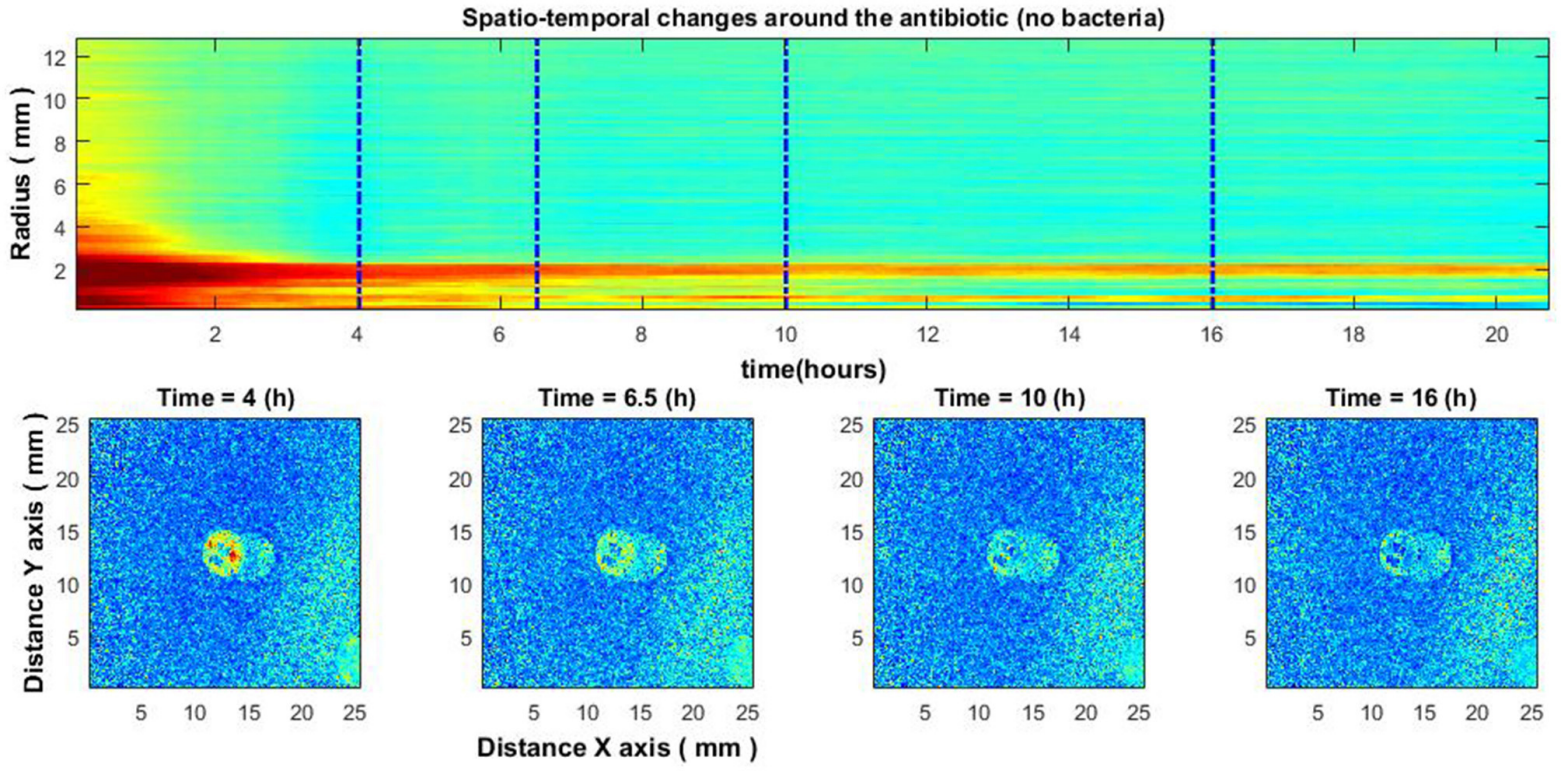
Experiment without bacteria. Spatio-temporal image of the area around the antibiotic AK 30 μg **(top)** and the spatial zone around the antibiotic at different times **(bottom)**.

**Figure 9:**
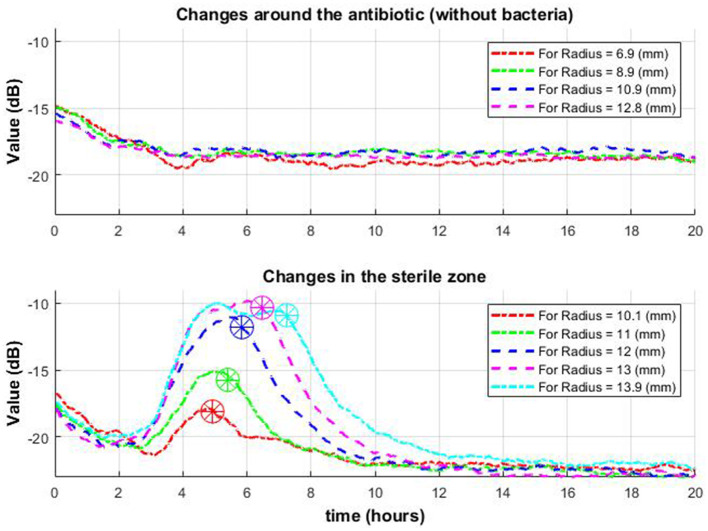
Change of signal envelope over time for several different radii around the antibiotic: without bacteria **(top row)** and with bacteria **(bottom row)**. On the bottom graph, it is observed that as it moves away from the center, the drop (which means the appearance of a zone of inhibition in this place) occurs later (stars in a circle).

The authors apologize for this error and state that this does not change the scientific conclusions of the article in any way. The original article has been updated.

